# Experimental Evidence of the Tonic Vibration Reflex during Whole-Body Vibration of the Loaded and Unloaded Leg

**DOI:** 10.1371/journal.pone.0085247

**Published:** 2013-12-30

**Authors:** Lisa N. Zaidell, Katya N. Mileva, David P. Sumners, Joanna L. Bowtell

**Affiliations:** 1 Sport and Exercise Science, London South Bank University, London, United Kingdom; 2 Sport and Health Sciences, University of Exeter, Exeter, Devon, United Kingdom; Northwestern University Feinberg School of Medicine, United States of America

## Abstract

Increased muscle activation during whole-body vibration (WBV) is mainly ascribed to a complex spinal and supraspinal neurophysiological mechanism termed the tonic vibration reflex (TVR). However, TVR has not been experimentally demonstrated during low-frequency WBV, therefore this investigation aimed to determine the expression of TVR during WBV.  Whilst seated, eight healthy males were exposed to either vertical WBV applied to the leg via the plantar-surface of the foot, or Achilles tendon vibration (ATV) at 25Hz and 50Hzfor 70s. Ankle plantar-flexion force, tri-axial accelerations at the shank and vibration source, and surface EMG activity of m. soleus (SOL) and m. tibialis anterior (TA) were recorded from the unloaded and passively loaded leg to simulate body mass supported during standing.  Plantar flexion force was similarly augmented by WBV and ATV and increased over time in a load- and frequency dependent fashion. SOL and TA EMG amplitudes increased over time in all conditions independently of vibration mode. 50Hz WBV and ATV resulted in greater muscle activation than 25Hz in SOL when the shank was loaded and in TA when the shank was unloaded despite the greater transmission of vertical acceleration from source to shank with 25Hz and WBV, especially during loading. Low-amplitude WBV of the unloaded and passively loaded leg produced slow tonic muscle contraction and plantar-flexion force increase of similar magnitudes to those induced by Achilles tendon vibration at the same frequencies. This study provides the first experimental evidence supporting the TVR as a plausible mechanism underlying the neuromuscular response to whole-body vibration.

## Introduction

Whole-body vibration (WBV) exercise has been shown in some cases to acutely increase muscle activation during exposure [1,2], lead to post-activation potentiation [3,4], and improve muscular performance [5,6]. Although not fully understood, the factors that govern the WBV-response can offer insight into the use of this form of exercise and its implications within the fields of fitness, health, and rehabilitation. 

Various neural mechanisms have been implicated in WBV-induced increased muscle activity. Despite the lack of direct evidence, the most frequently cited mechanism underpinning the WBV response is a reflex muscular contraction termed the tonic vibration reflex (TVR) that occurs during direct vibratory musculo-tendinous stimulation [7,8]. WBV is delivered through the soles of the feet and through the body rather than directly to the musculature, with frequencies up to 50Hz typically employed [1,7]. However, vibration frequencies in the region of 100Hz are suggested to excite muscle spindles and enhance activation of Ia afferents resulting in recruitment of higher threshold motor units with synchronous firing with vibration frequency [9,10] and a gradual development in muscle activity and/or joint torque that is typically used to determine the expression of the TVR [11,12]. The application of vibration to muscle involves transmission of the stimulus through the skin and often through anatomical segments, therefore skin and tendon receptors may also be activated and provide sensory signals to the somatosensory cortical areas of the brain. Whilst the TVR could account for increases in muscle activity seen during WBV, other mechanisms such as voluntary muscle conditioning contractions [13] and increased muscle temperature may contribute to any performance effects seen after WBV, with muscle-tuning and neuromuscular factors of both peripheral and central origin also proposed as candidate mechanisms [3,8].

Continuous leg muscle activity is required to maintain upright posture against gravity even during quiet standing [14], and sensory stimulation of the plantar forefoot zones by vibration has been shown to disturb postural regulation with whole-body tilts and shank muscle responses possibly occurring through integrative mechanisms involving supraspinal structures [15]. Hence, when standing on a vibrating platform, the postural instability induced by plantar-surface vibration may evoke modulation of spinal reflexes by supraspinal influences on the α-motoneuron pool. This may give rise to increased phasic muscular activation rather than a tonic contraction in response to vibration stimulation. In addition, direct vibration of a relaxed antagonist muscle has been shown to suppress TVR in agonist via the spinal mechanism of reciprocal inhibition; during WBV however, both antagonist and agonist muscles are vibrated which has led to doubts concerning the evocation of the TVR [8]. It is therefore challenging to isolate the supraspinal, spinal and peripheral mechanisms that contribute to any WBV-induced neuromuscular changes during unrestrained upright standing. As such, experimental evidence demonstrating the occurrence of the classic TVR during WBV is lacking. This study was therefore designed to address such issues, and in controlling for both postural instability and voluntary muscle activation, we hypothesised that WBV of relatively low frequencies can induce TVR. 

## Methods

### Ethics statement

This study was approved by the London South Bank University Research Ethics Committee. Experiments were conducted in accordance to The Code of Ethics of the World Medical Association (Declaration of Helsinki), printed in the British Medical Journal (18th July 1964). Each participant provided written informed consent before participating in the experimental trials. 

Eight healthy and recreationally active males participated in this study (age: 34 ± 7 years, height: 174 ± 10cm, weight 80.8 ± 24.3kg). Participants attended a familiarisation session a week before the main trial which involved brief (<10s) exposure to vibration, experience of leg loading, and a briefing of the experimental protocols. In a single experimental trial, participants performed 8 conditions each lasting 70s (separated by 2min of seated rest); systematic rotation was employed to counteract order effects. With participants in a relaxed stable seated position, the soles of feet were placed on the vibration platform surface (Fitvibe Medical, Gymna Uniphy, Germany), and with the thigh parallel to the floor, ankle-joint angle was controlled to mimic that seen during 30° squatting (internal knee angle) – measured during the familiarisation session. 

The right-leg was stimulated with either whole-body (WBV) or Achilles tendon vibration (ATV) at 25- and 50Hz (1.5 mm peak-to-peak amplitude). The left-leg was also placed on the platform but was not positioned to contribute to any measured force output. To simulate lower-limb loading during standing, the shank was externally loaded with ~45% body mass - equivalent to the body mass supported by the shank [16] via a custom-made loading rig positioned on the thigh just above the knee during 4 conditions (loaded; L: 25HzWBV, 50HzWBV, 25HzATV, 50HzATV). The rig was positioned in the same fashion but without a load for the other 4 conditions (unloaded; UL: 25HzWBV, 50HzWBV, 25HzATV, 50HzATV). Participants were given an arithmetic-based task [17] to divert attention and minimise the confounding effects of voluntary muscle control on TVR development. 

The head of the tendon vibrator (Unilab, England) was aligned with the mid-point of the Achilles tendon (between the calcaneous and *m*. triceps surae insertion) and in light contact with the underlying skin when the vibrating arm was at its shortest length. The device was secured to the shank using strapping and its weight was supported by an external frame. 

A load cell (MCL, RDP Ltd., Wolverhampton, UK) was incorporated into the custom-built loading rig and used to measure plantar-flexion force (PFF; N) resulting from vibration-induced muscle activation. Electrical activity of the m. tibialis anterior (TA) and m. soleus (SOL) was recorded with surface EMG sensors (DE2.1, DelSys Inc., USA) positioned over the muscle belly in accordance with recommendations for Surface Electromyography for the Non-invasive Assessment of Muscles (SENIAM; [18]). Reference gel-pad electrode was placed over the patella. To reduce motion artefacts, the electrodes and cables were secured to the skin using adhesive tape. Tri-axial accelerometers (ACL3000, Biometrics, UK) were fixed to the lateral side of the leg at the shank centre of mass [16] using adhesive tape and self-adherent elastic wrap, and to the tendon vibrator head and the vibration platform surface to measure accelerations along the vertical (Ve), and horizontal (medio-lateral: M-L; anterior-posterior: A-P) axes. 

EMG, accelerations, and PFF data were simultaneously recorded via an analog-to-digital converter (CED 1401power, Cambridge, UK) using Spike2 software, with EMG sampled at 2kHz, acceleration sampled at 1kHz, and PFF sampled at 200Hz.EMG signals were amplified by x1000 (Bagnoli-8, DelSys Inc., USA) and high-pass filtered with a 20Hz cut-off frequency. 

**Figure 1:**
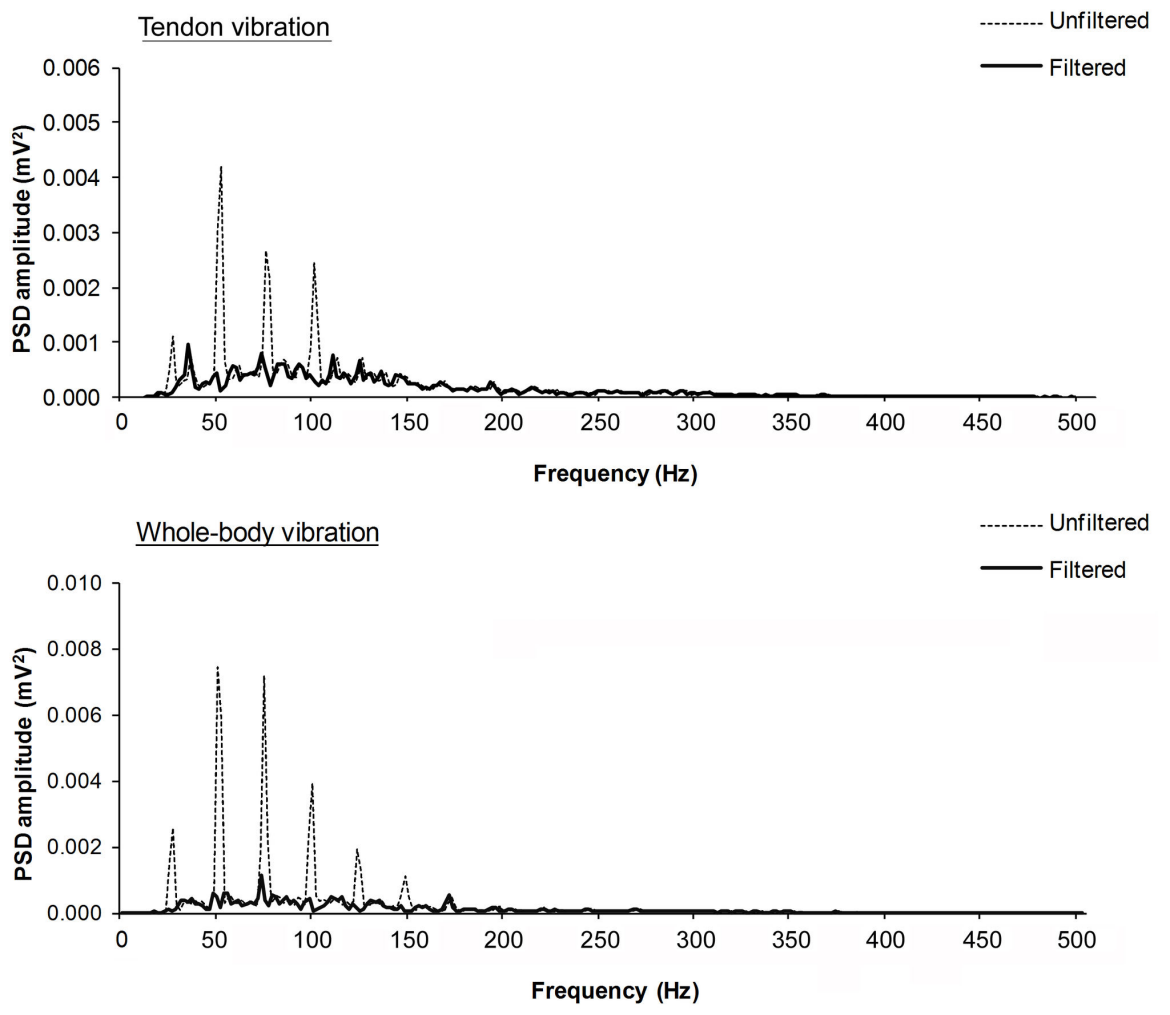
Example of the power spectral density (PSD) distribution of the EMG signal recorded from the *m*. **soleus during either tendon vibration or whole-body vibration (25Hz, 1.5mm) before (unfiltered, dashed line) and after (filtered, black line) applying the ‘spectral smoothing’ procedure to attenuate vibration artefacts**.

The vibration-induced artefacts in the raw EMG were attenuated with a spectral smoothing procedure [19]. Based on the cyclical nature of the vibration signal, the artefact superimposed to the EMG activity can be represented as a mixture of sinusoids of frequencies and amplitudes corresponding to the main and sub-harmonic vibration frequencies. Spectral analysis of the raw EMG signal was performed by Fast Fourier Transform with a block size of 1.024 s using a Hanning window function and the spectral distribution was presented between 0 and 1000 Hz in 512 bins at a resolution of 1.953 Hz. The presence of motion artefacts was confirmed by visual inspection and data were subdivided into blocks of one period of the sinusoidal waveform to be removed. The wave amplitude and phase in each block were determined by multiplying the source data by a sine and a cosine wave of the removed frequency, which was then subtracted from the original signal on a cycle-by-cycle basis. Before subtraction, the amplitude of the removed sinusoid was corrected by a ratio calculated from the power spectral density (PSD) of the signal to reflect the proportion of the signal power at the removed frequency above the average power of two neighbouring frequencies on each side of the spectrum. This procedure enabled the removal of excessive energy at the fundamental frequency of vibration and its harmonics whilst allowing some of the frequency components to remain in order to preserve the integrity of the physiological EMG signal (Figure 1).

The root mean square (RMS) amplitudes of PFF, TA and SOL EMG signals were calculated over 5s epochs to represent 10 time-points (t1-t10) during the middle 50s period of stimulation (uniform vibration stimulus). EMG data were normalised to a 5s epoch (t0) immediately before vibration onset. Vibration-induced accelerations at the WBV platform, tendon vibrator head, and shank were quantified as RMS units of gravity (g) calculated from t10. The source-to-shank vertical acceleration ratio was calculated to assess the vibration stimulus transmission to the shank.

Data were not normally distributed (Shapiro-Wilks) therefore Friedman’s test for repeated measures was used for all comparisons (SPSS 18.0, Chicago, Illinois). PFF and EMG amplitudes were statistically analysed for significant changes over time for each condition. The effects of vibration frequency (25- and 50Hz) and mode of application (ATV and WBV) on PFF, EMG, and acceleration data were analysed using the final (t10) values for each loaded and unloaded condition. An alpha level of 0.05 was set to establish statistical significance of differences.

## Results

**Figure 2:**
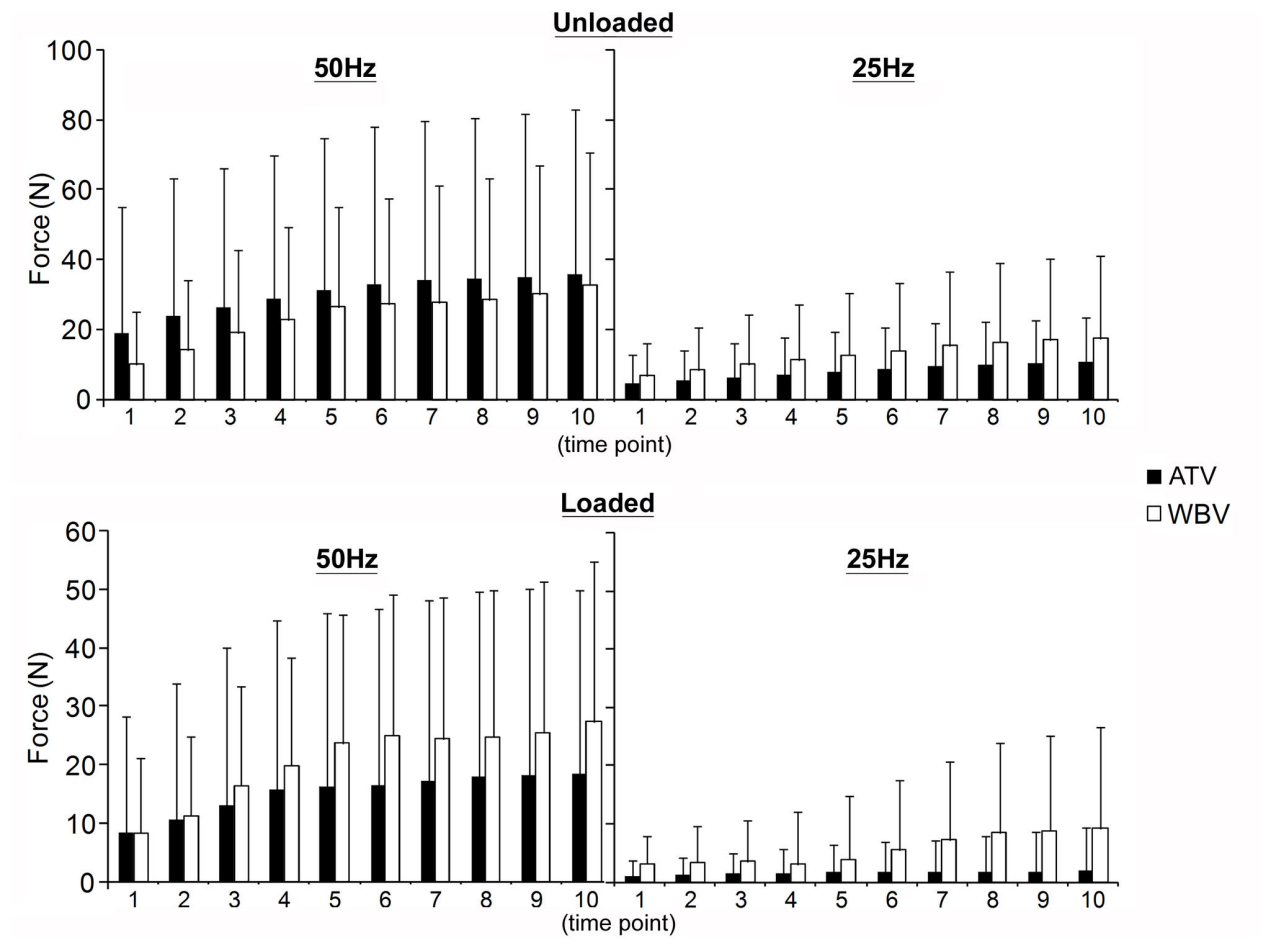
Plantar-flexion force during vibration. Changes in plantar-flexion force (mean ± SD) over 10 time-points (1-10) during Achilles tendon or whole-body vibration at 50Hz and 25Hz. L: loaded; PFF: plantar-flexion force; UL: unloaded shank; ATV: tendon vibration; WBV: whole-body vibration.

PFF was not different between the modes of vibration (WBV *vs.* ATV) but increased in a frequency dependent fashion (L: p=0.01; UL: p=0.003). PFF increased over time during 50Hz vibration of both loaded ATV (p<0.01) and WBV (p<0.01) of the shank and unloaded WBV (p<0.01) but not unloaded ATV (p=0.14). 25Hz vibration of the unloaded shank increased PFF over time (ATV: p<0.01; WBV: p<0.01) but not when shank was loaded (WBV: p=0.96; ATV: p=0.59; Figure 2). 

**Figure 3:**
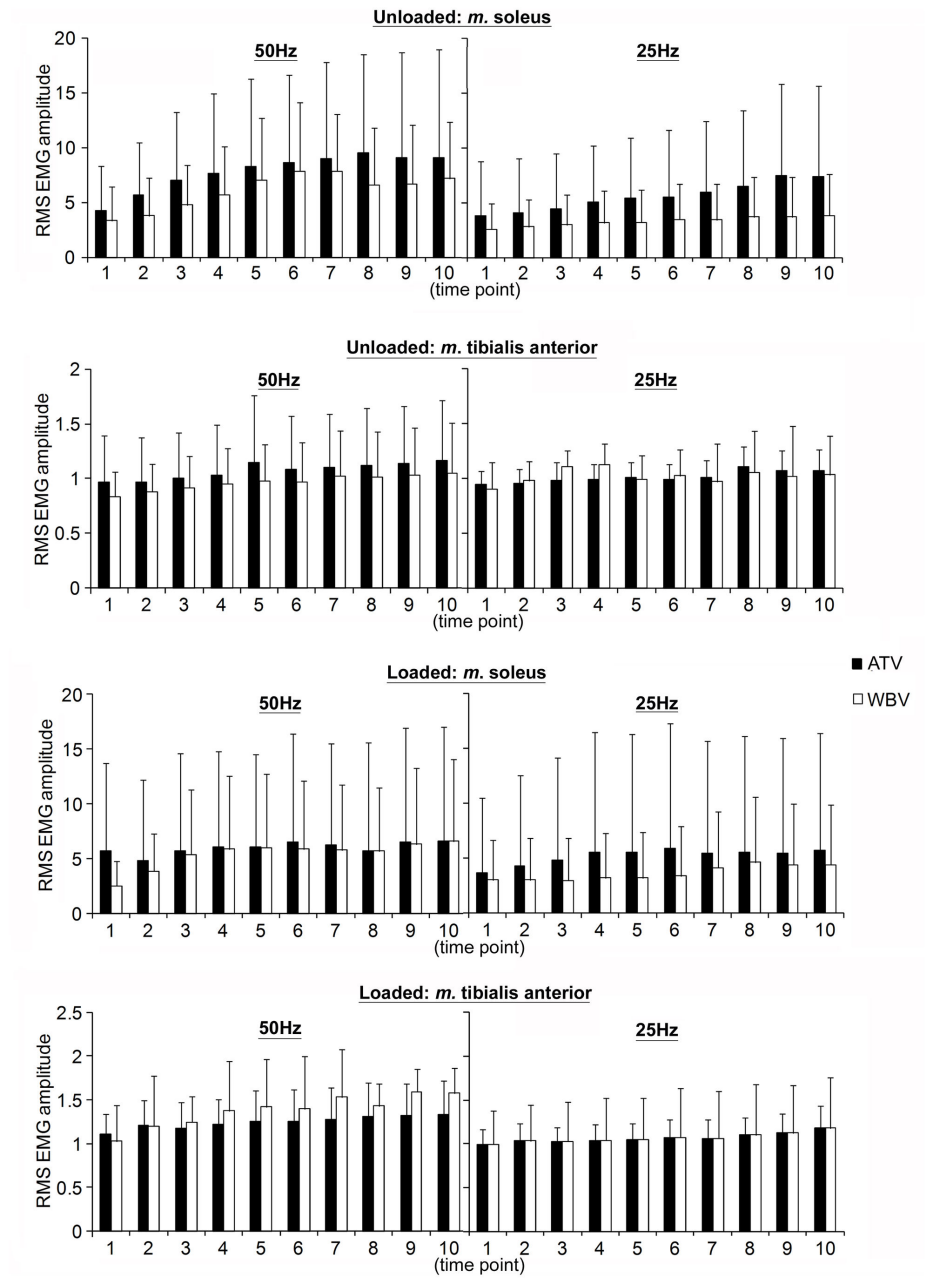
EMG amplitude during vibration. Normalised RMS EMG amplitude (mean ± SD) of the *m*.tibialis anterior and *m*. soleus during unloaded and loaded shank for each condition during whole-body vibration and tendon vibration. L: loaded; UL: unloaded; ATV: tendon vibration; WBV: whole-body vibration.

There was no effect of vibration mode on EMG activity. SOL muscle activation increased in a frequency dependent fashion for the loaded (p=0.04) but not unloaded (p=0.13) condition. Frequency effect was shown for the TA only when unloaded (L: p=0.13; UL: p=0.05; Figure 3).Both normalised SOL and TA EMG activities increased over time in all unloaded (all p<0.01) and loaded (all p<0.01) conditions (50HzATV, 25HzATV, 50HzWBV, 25HzWBV; Figure 3). 

**Figure 4:**
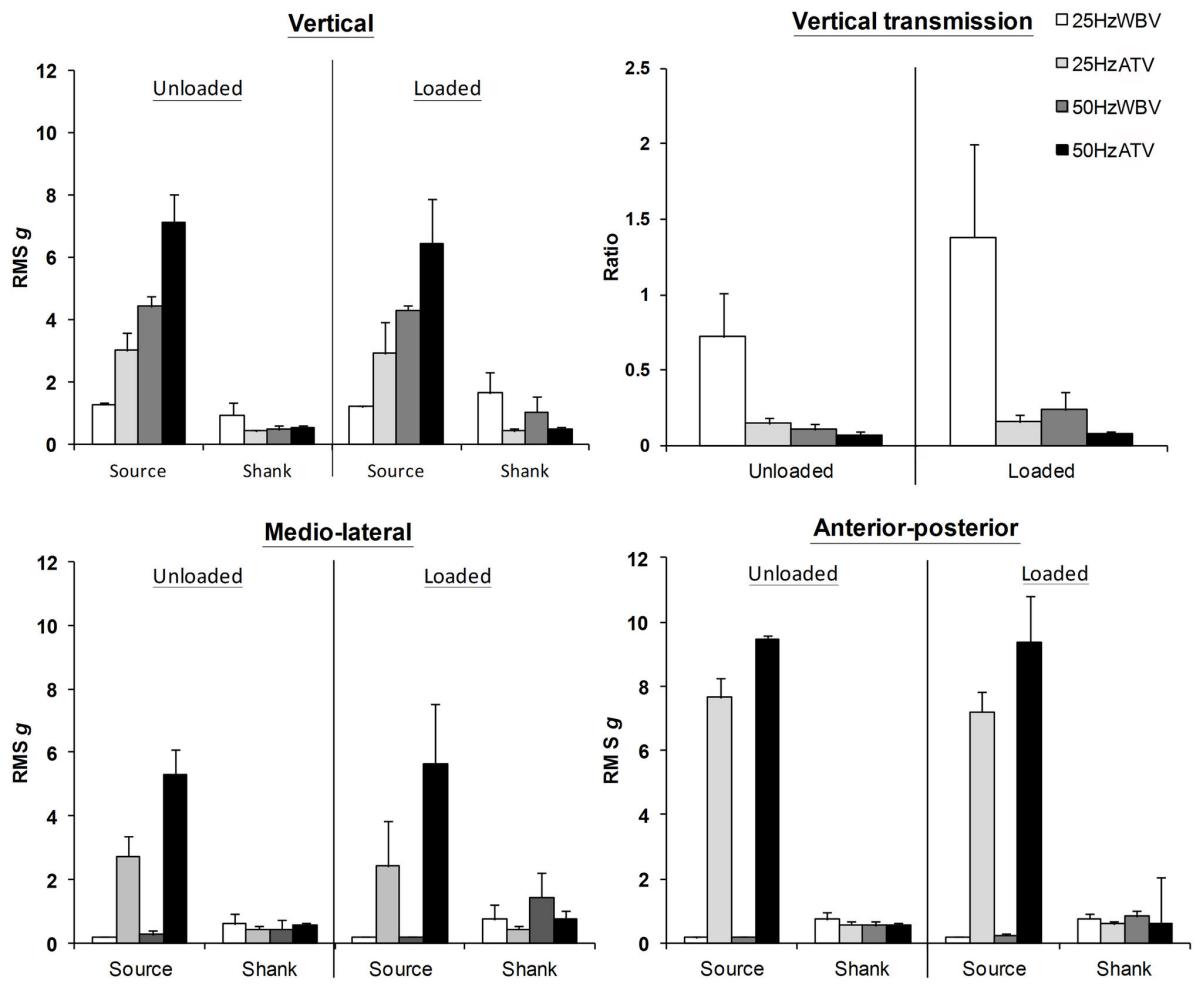
Tri-axial accelerations and transmission. Population mean (± SD) root-means-square accelerations registered along vertical, anterior-posterior, and medial-lateral axes at source and shank during unloaded and loaded shank conditions, and transmission ratios of vertical acceleration from the vibration source to the shank level. A-P: anterior-posterior; L: loaded; M-L: medio-lateral; ATV: tendon vibration; RMS: root mean square; UL: unloaded; V: vertical; WBV: whole-body vibration.

Greater acceleration was registered along all three axes (Ve, M-L, A-P) at the ATV head than the WBV platform (all p≤0.001); and at 50- vs. 25Hz vibration (p≤0.05) for both loaded and unloaded conditions (Figure 4). Greater Ve shank acceleration was registered during WBV than ATV for the loaded (p=0.01) but not unloaded (p=1.0) condition, with no significant difference between that produced by 25Hz and 50Hz vibration (L: p=1.0; UL: p=1.0). A-P and M-L shank accelerations were not dependent on the mode of vibration application for the unloaded condition but in the loaded condition WBV produced greater levels than ATV (A-P: p=0.01; M-L: p=0.05). Vibration with 50Hz frequency induced greater horizontal accelerations only in the M-L direction when the shank was unloaded (p=0.02). 

Transmission of Ve acceleration from the vibration source to the shank (Figure 4) was dependent on both vibration frequency and mode of application with transmission higher at 25Hz than 50Hz (p≤0.005) and during WBV than ATV (p≤0.001). 

## Discussion

Despite being commonly cited as the mechanism governing the WBV-response, to date the tonic vibration reflex has only been shown to occur in response to high-frequency direct muscle/tendon vibration [7,8]. Using surface EMG and force output recording, this study provides the first direct evidence that low frequency whole-body vibration is able to elicit classic TVR responses in lower limb muscles when delivered during relaxed sitting. WBV of the unloaded and passively loaded leg induced parallel increases in plantar flexion force and *m*. soleus activation – likely reflecting summation of Ia afferent activity via successive stretch-reflex cycles - indicative of TVR [12]. Furthermore, the strength of the WBV-induced TVR in lower limb muscles was similar in magnitude to that induced by Achilles tendon vibration at the same frequencies. 

WBV has been shown to produce non-negligible mechanical artefacts to the electromyogram which can increase EMG amplitude by 40% in some cases [20]. Where filtering methods have been applied for the removal of vibration-induced artefacts, increases in muscle activation during WBV are still evident [2,21,22]. In the present study, vibration-induced motion artefacts were observed on the EMG recordings and a spectral smoothing procedure (Figure 1) was applied to remove these. Thus, with vibration-induced motion artefacts accounted for, the increased EMG activity observed in this study and accompanied by plantar-flexion force production is likely to result from reflex muscle contractions entrained by the vibration stimulation.

Vibration frequencies up to 50Hz are typically employed for WBV investigation, with the frequency of vibration influencing the degree of muscular activation during squatting exercise [20,21]. Most studies investigating WBV exercise protocols adopt frequencies between 15-35Hz - a range where, to our knowledge, there is no published evidence of the existence of TVR. The vibration frequencies chosen for investigation in the present study were selected on the basis that 50Hz tendon/muscle vibration can induce TVR [11] and previously, we have found that within the frequency range 20-35 Hz, 25Hz (1.5mm peak-to-peak) vertical WBV produces the greatest stimulation of shank musculature during shallow isometric squatting [23]. Fratini et al. [20], have also found similar results for m. rectus femoris activation during deeper squat posture and similar vibration amplitude (1.2mm peak-to-peak vertical WBV) after accounting for the influence of vibration-induced motion artefacts.

Motor unit firing is suggested to synchronise to the frequency of vibration and its sub-harmonics [10] – a phenomenon that may underlie greater muscle activation during higher frequency vibration [7]. We also observed greater increases in PFF with 50Hz vibration stimulus than 25Hz. Higher frequencies (up to 150Hz) are reported to be more effective for TVR evocation during direct vibration application [10], hence the lack of evidence for eliciting TVR in muscles using frequencies below 40Hz. Further, during WBV, the difficulty of isolating the classic TVR response from other modulatory mechanisms involving postural reflexes and/or voluntary muscle contractions during upright standing exercise also presents a major challenge for demonstrating TVR. Here, the increase in m. soleus activation was not different between frequencies when the shank was unloaded; when the shank was loaded however, a frequency dependency existed with greater RMS EMG amplitude seen during 50Hz. The opposite was true for the m. tibialis anterior where the higher frequency of vibration induced greater muscular activity when the shank was unloaded but not loaded. This may suggest that being antagonistic to each other, the activation of these two muscles during vibration stimulation is dependent on the effects of Ia inhibitory interneurons [24], or may be due to modulation of the biomechanical properties of the shank that may arise with loading such as muscle/joint stiffness, as well as differences in load afference and receptor stimulation. 

Previously, direct high-frequency vibration of muscle or tendon has been proposed to be a pre-requisite for TVR evocation [8], with its strength influenced by the location of vibration stimulation, the initial length of the vibrated muscle, level of central nervous system excitability, and vibration parameters [25]. Muscle activation induced by WBV stimulation was thought unlikely to occur via TVR due to the involvement of simultaneous indirect vibration of agonist-antagonist muscle complexes during exposure [8]. We investigated two antagonist postural muscles – the m. soleus and the m. tibialis anterior. Via inhibitory interneurons, the contraction of antagonist muscles is reduced when the agonist is directly vibrated [26]; here however, a WBV-induced activation of the SOL was demonstrated in all experimental conditions which coincided with TA activation. Thus, simultaneous low-frequency vibration of agonist-antagonist pairs during WBV may attenuate rather than fully inhibit TVR. Of the two muscles, greater vibration-induced muscle activation was seen for the SOL which occurred in parallel with ankle plantar-flexion force development. The flexed ankle-joint position adopted in this study may have facilitated the greater activation of the SOL to bring about plantar-flexion since the strength of TVR is enhanced in lengthened muscles during isometric contraction - presumably due to the enhanced response of Ia spindles that are sensitive to muscle stretch [27]. During ATV - when only the tendon to m. triceps surae was directly vibrated – TA was also active, which may have resulted from an antagonistic vibratory response that arises with kinaesthetic illusory movement [26]. Co-contraction for joint stabilisation may also necessitate TA activation especially during the loaded shank conditions [28]. 

WBV training is often performed whilst in standing posture on the platform, during which tonic activity of anti-gravity muscles occurs to provide a stabilising force for maintenance of postural equilibrium [14]. TVR has been shown to occur both in relaxed [11] and active [10] muscles during locally applied high-frequency vibration. Hence, in an effort to gain insight into the mechanisms of WBV during standing exercise, we investigated the shank musculature not only when relaxed and unloaded but also when passively loaded with an external weight to approximate the natural loading on this segment during standing. In the present study a TVR response was evoked by WBV, as evidenced by the gradual increase in SOL EMG activity in both loaded and unloaded conditions. Similarly, it has been shown that background muscle activation and gravitational loading are not necessary for evoking neuromuscular activity of the m. soleus and gastrocnemius in response to mechanical stimulation of the plantar-surface of the foot [29]; modulation of spinal motoneuronal excitability during standing WBV without voluntary contraction of the leg musculature has also been recently reported [30]. Here we observed a parallel increase in PFF and muscle activation in all conditions apart from when the leg was loaded and vibrated at 25Hz (WBV and ATV) – perhaps due to lower muscular activation with this frequency combined with the requirement to overcome external loading of the leg. Differences in TVR response during unloaded and loaded conditions, particularly those seen with ATV, may result from modulation of spinal excitability that may be dependent on location of stimulation [30]. Alterations in plantar-pressure sensations during loading may have inhibitory effects on spinal reflexes that arise from tendon stimulation, whereas facilitation could result from plantar-cutaneous afferent excitation [30]. Thus, the stimulation of different types of receptors (cutaneous, foot/ankle, tendon, muscle) resulting from both vibration and loading may modulate the TVR, with the degree of receptor excitation dependent on the mode of vibration (ATV or WBV), along with frequency and amplitude of vibration. We have previously demonstrated modulation of corticospinal excitatory input to TA and SOL during WBV [19], however, whether supraspinal mechanisms activated by postural instability during standing WBV may additionally modulate the TVR cannot be determined here. 

During tendon vibration, there was a marked reduction of vertical, anterior-posterior, and medio-lateral acceleration at the shank indicating that acceleration transmission from the vibration source was poor. Despite lower Ve acceleration measured at the WBV platform compared to that at the tendon vibrator head, transmission to the loaded shank was greater with WBV. Furthermore, although 50Hz imparted greater tri-axial accelerations at both sources of vibration than 25Hz, shank Ve accelerations were not different between the two frequencies. Compared to 50Hz, the transmission of Ve acceleration from source to shank was greater with 25Hz where an amplified acceleration response was observed, especially when the leg was loaded. As observed previously [23,31], WBV of 25Hz is more effective at delivering vibration to the musculature of the shank, probably due to more efficient damping of higher-frequency vibration; yet interestingly muscle activation was higher at 50Hz. This may reflect stronger muscular facilitation via supraspinal and spinal (e.g. TVR, motor unit recruitment and synchronisation) at higher vibration frequencies.

Non-linear muscle activation responses that are sometimes observed during WBV of low–frequency may be explained by resonance properties of tissues and vibration damping [20,32]. Amplification of vibration acceleration has been shown to occur between 10-40Hz at the ankle [33]; 20Hz WBV also induced greater shank acceleration compared to 40Hz [31]. Such vibration amplification during low frequency WBV, presumably by the structures of the foot and ankle, may favour the use of WBV over tendon vibration for muscular stimulation or for targeted transmission to skeletal structures. Amplification of vibration-induced soft-tissue acceleration is linked to natural resonant frequencies of muscle tissue and correlated to muscle EMG activity [20] - although such findings do not entirely agree with those of the current study. Here, although greater transmission of vertical acceleration from the source to the shank was seen with 25Hz, the highest muscle activation was not necessarily shown to occur at this frequency - m. soleus activation of the unloaded shank was not different between frequencies, whilst that seen when the shank was loaded was higher during 50Hz vibration. Plantar-flexion force production was also greater with 50Hz versus 25Hz suggesting that a stronger TVR was seen with the higher frequency of vibration. Thus, during WBV exposure, when the lower limbs support body mass, for example during upright standing or squatting, higher vibration frequencies may be required to maximise muscular activation via central neural mechanisms. Non-linear responses to WBV frequency have also been reported [20], which may depend on variations in exercise posture, body composition, interrogated muscles, and propagation of the vibration stimulus through the lower limb. Regardless, that the TVR was evoked in the current study during stable posture, both when the shank was unloaded and passively loaded, suggests that WBV can induce muscular activity via both peripheral mechanisms and spinal reflexes independent of postural control mechanisms. 

## Conclusions

The tonic vibration reflex response can be evoked in both passively loaded and unloaded relaxed shank muscles during seated whole-body vibration of relatively low frequencies (25- and 50Hz) when applied through the plantar-surface of the foot. WBV therefore offers a practical method for training relaxed musculature which has applications to health and rehabilitation (e.g. injury, immobilisation, bed-rest, micro-gravity).

The transmission of vibratory acceleration along the leg was superior when applied via whole-body-compared to tendon-vibration, especially at 25Hz frequency. This is likely due to modulation of the imparted vibration by the foot and ankle structures, and therefore WBV at lower frequencies does not seem to limit the evocation of TVR. Higher frequency vibration (50Hz *vs.* 25Hz) should be chosen for greater muscle activation. Furthermore, TVR is operative during WBV when antagonist musculature is simultaneously vibrated; therefore this study offers experimental evidence that supports the TVR as one of the mechanisms underlying the neuromuscular response to WBV. 
